# Mr. Brodie on Calculous Disorders
*Continued from the last page of the review department.


**Published:** 1831

**Authors:** 


### LXII.

### MR. BRODIE ON CALCULOUS DISORDERS.*

WE give the following lectures in the form in which they were taken by a gentleman of Mr. Brodie's class. They will be found to correspond pretty accurately, no doubt, with those in our contemporary, the Medical Gazette, in which they have not yet appeared. We have done this in order that the subject might be completed in one Quarterly Number of this Journal, and that our readers might at once be in possession of what we cannot hesitate, for an instant, to designate as the most valuable series of practical lectures on the diseases of the urinary organs, which have ever been given by any lecturer to any class. Having now completed them we need say no more than that they speak their own best eulogy. A great number of valuable facts are communicated in a plain style without show or parade, and it would be well for surgery and for science if writings of this description were more numerous than they are. We trust that Mr. Brodie will long continue to favour the profession with the results of his extensive experience and keen observation. He must remember that he owes us yet the sequel of his former paper on "Injuries of the Head," and we hope, and venture to expect, that he will give more, although more has not been promised.

---

* Continued from the last page of the review department.

### Calculus in the Female Bladder.

This is comparatively rare. The precise proportions of men and women affected, it would be rather difficult to ascertain, but I should say roundly about 1 woman to 15 men. The *symptoms* in women are :—frequent inclination to make water—pain in the extremity of the urethra—urine tinged with blood after exercise—urine undergoing changes, leading to the deposition of triple phosphate, or even phosphate of lime.

Sometimes there is in women a sort of *natural cure*.

In a female at St. George's Hospital a stone of some size ulcerated into the vagina, and a fistulous opening was left. In another case the kidney looked, as I have already mentioned, as if it were converted into fungus hæmatodes.

### Treatment of Calculus in the Bladder.

*Detection of the Stone.* The first thing is to find the stone. The symptoms generally inform you of its presence, but sometimes they mislead. You must examine the bladder by what is termed *Sounding*. The iron sound ought to lie loose in the urethra, filling but not stretching it. The patient should generally lie upon his back, with his shoulders a little elevated ; sometimes it is better for him to stand. If the stone is large the bladder may be full ; if small, it should be more empty. If the calculus be small, explore all the parts of the bladder ; and if the symptoms be well marked, don't necessarily conclude that there is no stone because you cannot find one. Sometimes a gum catheter will detect a stone, when an ordinary sound will not. Introduce the gum catheter without the stilet, the patient standing, and the bladder being as full as possible ; as the last drops of urine come away, the stone falls against the gum catheter ; this is a very valuable method. Sometimes an examination with the finger in the rectum is useful, particularly in children, when the stone is of middle size. In adults it is of little service, unless the stone be large.

*Means of learning the size of the Stone.* If you try with care you may form a pretty accurate idea of the size of the stone. If this be not of long formation, it is usually composed of lithic acid or oxalate of lime. If the urine is alkaline, the last layer must be composed of the phosphates, and then the growth is more rapid. If the calculus has existed for very many years, you may conclude that it is a large one. The most precise knowledge is obtained by *measuring* the size of the stone with a sound. When there is water in the bladder, you may push the stone out of the way with the sound, and the force necessary to displace it may give you some notion of its comparative weight and size.

When a stone has been carried into the bladder, it may be washed away by the stream of urine, but sometimes this is prevented by an enlarged prostate. Persons pass kidney calculi for many years till they grow old, and the prostate becomes enlarged. Such a patient should make water in the attitude of leaning forward, by which the projection of the prostate is in some measure counteracted. The plan is good in theory, and, as I think, in practice. A patient whom I directed to make water in this way soon became relieved of a small stone which had lodged for some weeks previously in the bladder.

*Dilatation of the Urethra—Diluents.* When the stone is larger than the urethra, it cannot of course pass through it. But you may dilate the urethra, and that without delay. I introduce on one day a large sound or bougie, on the next day a larger one, and so on, dilating the urethra as far as I can. Then I give diluents, sometimes diuretics, as the spiritus juniperi compos. to procure as great a torrent of urine as possible. It is a good plan to introduce every day or every other day a full sized bougie into the bladder, and let it lie in the urethra. Then the patient drinks an abundance of diluents till the bladder is much distended, and there is a violent nisus to make water. Then let the patient lean forward and strain to pass his urine; withdraw the instrument, the urine comes away in a gush, and probably the stone with it. This plan was first suggested to me by a patient.

*Urethra Forceps.* If the former means fail, you may generally extract a small stone by the urethra-forceps, when there is no material alteration of the bladder preventing it from containing a fair quantity of urine, nor a large tumour of the prostate. This is one of the greatest achievements of modern surgery, due to Sir Astley Cooper and Mr. Weiss. In employing the urethra-forceps the bladder should hold ℥vj. or ℥viij. of water; indeed, many inject warm water into it through the catheter. It is generally prudent to ascertain first, by a sound, where the stone lies. Then introduce the forceps, lightly feel the stone, open their blades, and try to seize it; much force is not required. Sir Astley has removed a great many stones from the bladder with this instrument, and I have successfully employed it in many instances. The neck of the bladder admits of great dilatation, the urethra of less, so you may seize a stone of considerable dimensions by the forceps, bring it into the urethra, and extract it from that by a slight modification of an operation.

*Case.* A gentleman had stone in the bladder. I seized the stone, and drew it down for some way into the urethra, but then I could get it no farther. I seized the forceps with the left hand, made the stone project in the perinæum, cut down upon it, and extracted it through the wound. I repeated this operation once or twice without any assistance. The wound healed readily, a gum catheter being kept in the bladder after the operation.

Be cautious how you perform this operation anterior to the scrotum.

*Case.* A gentleman came to me with symptoms of stone in the bladder. I laid him down, felt the stone, brought it with the forceps anterior to the scrotum, and, not being able to get it farther, I made an incision on it there, and cut it out. But the urine afterwards gravitated into the scrotum, and troublesome abscesses succeeded. I would, in future, only cut on a calculus behind the scrotum. I think we might improve much upon this operation.

I have already explained to you, that if you introduce a gum catheter, and draw off the contents of the bladder, where there is a small calculus, it very frequently happens, as the last portion of the urine flows, that the calculus is thrown down, as it were, on the end of the instrument. Then, it occurred to me, that if a catheter could be made to open like a pair of forceps, the calculus would very probably fall into it; that if it did not do so at one time, it would do so at another time, and that thus it might be extracted without searching and irritating the bladder—with little or no pain to the patient, and little or no trouble to the surgeon. With these impressions on my mind, I contrived the instrument which I now shew you. It is a pair of forceps with two blades, the opposite surfaces of which are made rough, like a rasp or coarse file. They open by withdrawing a tube, which encloses them, on the principle of one kind of bullet forceps, or of the French lithontriptic instrument. But the forceps are themselves a hollow tube, so that whenever the blades are separated, they answer the purpose of a catheter; allowing the urine to flow out of the bladder. Since this instrument was constructed, I have had only one opportunity of employing it, and that very lately. A gentleman consulted me with slight irritation of the bladder. I examined the bladder with an iron sound, and detected in it a very small calculus. I then dilated the urethra to its utmost extent. This was easily accomplished, but the calculus did not come away. I introduced Weiss's original urethra-forceps, but the stone eluded my search. I therefore introduced my new forceps, the bladder being full of urine; and the blades being expanded, of course the urine flowed. When the bladder was empty, I endeavoured to close the forceps, but I found that I could not do it. In fact, the stone was seized, and it was easily removed. It was of the size of a large pea; and the patient suffered not the smallest inconvenience from the operation.

*Solvents—Injection of the Bladder.* If the stone is too large to be extracted by the forceps, what are we to do? Can we dissolve it? The phosphates out of the body are soluble in the acids—the lithic acid calculus in alkalies. But only very strong alkalies will act on lithic acid, and the patient cannot take enough to dissolve it. How absurd. A patient takes alkalies internally—the urine becomes alkaline in consequence—alkalies are deposited round the lithic acid—and the stone grows the faster. Sometimes lithic acid unites with soda, and comes away in fragments. The patient thinks that portions of the stone are coming away, but in truth it is a *new formation.* A gentleman voided a whole wafer-box full of fragments of lithate of soda.

It may be asked if you would inject alkalies into the bladder;—it will not bear them strong enough. Nitric acid dissolves the lithic acid calculus out of the bladder, but it is too strong to be injected into that viscus. But it readily dissolves the phosphates, even when diluted, and it is a question with me whether it may not be usefully employed when the bladder is in too diseased a state for operation, and the stone chiefly composed of the phosphates.

*Case.* A gentleman had long had stricture of the urethra, chronic inflammation of the bladder, and secretion of ropy mucus streaked with phosphate of lime. Above all things in the world he was ordered to drink lime-water. I introduced an instrument and felt one stone, if not several. The stone was evidently not the original disease, but the consequence of the stricture, and I supposed that it was composed of the phosphates. Small fragments being passed proved that it was as I suspected. I had a gold catheter constructed, with two separate tubes in it. I introduced it into the bladder, and, throwing in a weak solution of nitric acid, through one tube, I let it run off by the other. I began with ♏j. of the acid to ℥j. of water, but I afterwards increased the strength to ♏iij. but the average strength that could be borne best was ♏ijss. I went on injecting, sometimes for half an hour at a time, when the liquid would contain the phosphates abundantly dissolved. The strong liquor ammoniæ, used as a test, readily throws them down. I believe that the nitric acid employed in this way only dissolves the phosphates, and the ammonia separates them. Well, under this treatment, the mucus of the bladder was very much diminished in quantity, and its character improved. Matters went on well, and at last the patient voided these two stones. (Specimens in the museum.) He could now walk into the street, but after a time the symptoms returned, and the patient died in the country of diseased kidneys.

*Case.* A gentleman had diseased prostate, and chronic inflammation of the mucous membrane of the bladder, with several small calculi. I continued to inject the bladder, off and on, for 12 months, got rid of the mucus, and at length the patient could go for six or seven hours without passing his urine.

It is evident then, from the foregoing facts, that there are some cases of stone, in which injection of the bladder does good. But more than this, the injection does good to the bladder itself.

*Case.* A patient had calculus in the urinary bladder, and the urine was so ammoniacal that the room stank. I injected the bladder—the urine became comparatively healthy—I cut the patient—and he did well.

When the stone is of small size, and entirely composed of the phosphates, this injection may dissolve it. Under other circumstances dissolution is out of the question.

### On the Operation of Lithotomy.

The calculus is to be extracted by incision of the bladder. You may cut into the bladder from above the pubes, or from the perinæum. The experience of surgeons is in favour of the latter method, but there are various modes of doing it. If a calculus is small, you may extract it by dilating the urethra. Now you can dilate the urethra to a certain extent, but no further, by bougies, and if the calculus is too large then to pass, you must dilate further by a cutting instrument. This you do by cutting in the perinæum, and incising the posterior part of the urethra.

And now of the method of performing the lateral operation of lithotomy.

*External Incision.* In the upper part of the perinæum the space for a cutting instrument is narrowed; in the lower it is broader. If you cut in the line of the raphé you endanger the rectum. You must incise the perinæum obliquely, gaining space and avoiding the rectum. This is the lateral operation.

*Points deserving of attention.* 1. Endeavour to have as little hæmorrhage as possible. I have myself lost one patient, and have seen two good lithotomists of this town each lose a patient. Believe me this is no bugbear. Some cut very freely, and they are in danger of hæmorrhage. Make no unnecessary incisions, and cut chiefly in the lower part of the perinæum, not too near the rami of the ischium and pubes, by which you avoid the branches of the pudic at their origin.

2. Make your incision so as to get room for the calculus in the lower part of the perinæum.

3. Don't make your incision at the neck of the bladder larger than is absolutely necessary; cut through the prostate, but *not beyond it.* This is of the very utmost importance. When you cut the prostate only, you cut no more than a solid substance, but when your incision extends beyond it, you cut into a loose cellular texture, sucking up the urine like a sponge. You know the symptoms that must ensue; the symptoms that ensue when the urethra gives way behind a stricture. Practically it is as you would expect.

*Case* 1. In 1810, a farmer was operated on by Sir Everard Home. The operation was done very rapidly; to a bye-stander it seemed remarkably dexterous. But, in opening the forceps, Sir Everard remarked that something gave way, as if a string was broken. In the night the patient was anxious, irritable. Next day the depression was greater—the pulse became rapid—the belly tympanitic. The patient died in 48 hours. On dissection, there was no peritonitis. The muscular and mucous coats of the bladder were lacerated half an inch or more, which was done, no doubt, when the forceps were opened. The cellular membrane was in a sloughy state, nearly as high as the kidney; in the lower part, about the incision, there was pus.

*Case* 2. In 1816, I operated on a child for the stone. The opening in the bladder was small, the stone large; I dilated the wound with a probe-pointed bistoury. In three days the child died, with low symptoms, tympanitic belly, &c. I had cut through the prostate into the cellular membrane, which was in a sloughy state.

A similar case occurred to Mr. Keate not a year ago.

Soon after the occurrence of the second case, I read Scarpa's work, in which he took the same views as myself. Since that I have had many cases which prove that an opening cannot be made into the loose cellular membrane with impunity.

*Steps of the Operation.* I generally introduce the staff before I bind the patient. The staff should be as large as possible, with a blunt extremity. Have four assistants, one for each leg, one for the staff, one for the head. The assistant holding the staff stands on the left side, his hand directed towards the right groin, the curve neither drawn too high nor depressed too low. The table should be about four feet six in height. The shoulders should be a little raised.

Then make your incision in the perinæum. Don't attempt to plunge the knife at once into the groove of the staff;—by so doing you endanger the bulb, and cut too high and too near the scrotum, which may become infiltrated with blood or urine. The usual point for commencing the incision above, is about an inch anterior to the anus, and nearly in the line of the raphé; then carry the incision freely down to the space between the anus and the tuber ischii. Always direct your attention to the staff—it must be your guide—if you neglect it all is random cutting.

Cut into the groove of the staff behind the bulb, just at the membranous part. If you cut too high, you endanger the arteries of the bulb, &c. and have to divide more soft parts, in order to get into the bladder. If it be a child you may go on with a scalpel; if an adult, this is not a good instrument to divide the prostate, the point being likely to hitch in the groove of the staff. Cheselden finished the incision of the neck of the bladder with a common scalpel, and then introduced a blunt gorget along the staff, withdrew the latter, and carried on the forceps along the gorget. This I believe, from Cheselden's own account, to have been his operation, though Dr. Douglas's account is different, and no doubt incorrect.

*Cutting Gorget.* Sir Cæsar Hawkins invented the cutting gorget. I cannot recommend it. It requires force in its introduction, and is apt to slip out of the groove of the staff into the space between the bladder and rectum. I have known this happen to two experienced surgeons, and what has occurred to one may occur to others. This accident occasions death in a few days, by sloughing of the cellular membrane. On the whole, the gorget is not a safe instrument.

*Mr. Brodie's Method.* In the adult I generally pass a scalpel, with a beak attached to it, along the groove of the staff into the bladder. *Never cut laterally*, with whatever instrument you employ; for you then don't know what you divide.

If the stone is large, why not cut both sides of the prostate? I then use a double-edged scalpel. Whichever you employ, get the beak into the groove of the staff, taking care that there is no cellular membrane, &c. in the groove. Then take the staff in the left hand, depress the handle, and at the same time the handle of the knife also, by which you cut low in the perinæum. Slide the knife along the groove, feeling the latter all the way. You reach the bladder—a *little* urine flows: you withdraw the instrument. Then pass your finger into the wound and feel the stone. If it be a child, or a thin perinæum, you can pass the forceps in at once; if not, introduce into the bladder, along the groove of the staff, a blunt gorget, larger or smaller according to the expected size of the stone. You have *nicked* the prostate; the gorget *splits* it up with ease, not dividing the firm capsule. The gorget being in the bladder, withdraw the staff. If possible, introduce the finger along the concave surface of the gorget into the bladder, and explore it. The best way to introduce the finger, is to turn the concave of the gorget towards the left side of the patient, by which you obtain room.

Have forceps of various sizes in readiness. Oil and warm them—introduce them carefully upon the gorget, and then withdraw the latter: feel for the stone with the forceps—open them cautiously and seize the stone. If the bladder is flaccid, there is little danger of lacerating it in opening the forceps; if it is contracted, there is more. Try to ascertain the size of the stone, and then endeavour to seize it in its short diameter. If it be a fusible calculus, it is very soft, and easily broken; be guided in this.

In withdrawing the forceps, turn the blades upwards and downwards, in the direction of the external wound, not transversely, for then the stone will catch against the prostate, and the latter acts as a valve preventing its withdrawal. Take time—use moderate force only. If the stone is soft, introduce between the handles of the forceps a piece of wood, or another pair of forceps, and push them against the joint of the forceps, whilst attempting to extract the stone. This will prevent you from crushing it.

If the stone is polished, you may be sure that there is another. Introduce your finger, or a bladder sound, in order to ascertain.

Sometimes a stone is lodged in some fold of the bladder, behind the pubes. Then introduce the forceps, open their blades transversely as wide as possible, and thus you dislodge the stone, which falls between the expanded blades. If the stone is lodged in the fundus, behind the prostate, employ curved forceps. Use small forceps for small stones, or a scoop; if the stone is broken into many fragments, wash out the bladder with a syringe.

I shall speak of hæmorrhage in the next lecture.

*After the Operation.* Place the patient in bed, with the shoulders a little raised, a draw-sheet under him, a bolster beneath the hams, and the knees *not* bound together. The urine always flows at first through the perinæum, and the first two or three gushes give much pain, which afterwards ceases. When there is a deep perinæum, it is a very good plan to introduce an elastic gum catheter into the bladder through the wound, and I think it lessens the danger of urinous infiltration.

*After-treatment.* This is generally very simple. If there is pain in the belly, have recourse to a fomentation and a few leeches. Aperient injections are better than purgatives—dressings are of no use, as they get sopped in urine. Where there is a fusible calculus, and especially where it has been broken, more attention is required. There has previously, in this case, been chronic inflammation of the bladder, and, therefore, there is a liability to another calculus. This is much more likely to take place where the calculus has been broken.

After two or three days you may introduce a gum catheter into the bladder, (when the calculus has been of the fusible kind) inject through it tepid water, and so wash out the mucus and sand which issue through the wound in the perinæum.

When the *prostate is enlarged,* I use a large silver catheter, with an eye four times the ordinary size, and inject tepid water daily for a length of time. The fragments escape through the large eye of the catheter, and I have thus saved patients from the re-formation of calculi. Besides this you must adopt the treatment already recommended for chronic inflammation of the bladder. It is an evil certainly for a *stone to break;* yet, if very large, I would purposely break it. The operation is thereby prolonged, but the neck of the bladder is not so much injured and wounded.

*Adherent Calculi.* You never meet with them except where the calculus is encysted. Even the latter is rarely met in operations, because encysted calculi occur in diseased bladders, and in cases not fitted for operation. I had one case of this kind in the hospital. The patient was a boy; sometimes I could feel the stone—sometimes I could not. I employed the cutting gorget. On cutting into the bladder I could at first feel no stone; but, having explored the interior of the bladder very carefully, I found, an opening above the pubes, and in it a calculus. I nicked the side of the cyst, and then could peel it from the stone, a portion coming away sticking to it. There was much constitutional disturbance, but the patient recovered.

### ON DEATH FROM LITHOTOMY.

I cannot too strongly recommend to you the study of the cases that prove fatal. It is from them that you may learn how to prevent other cases from proving so.

*Too free division of the prostate* is one cause of death. What are the consequences of this too free division? At first the symptoms are not well marked. There is some heat of skin, quickness of pulse, want of sleep; but the more characteristic symptoms seldom begin later than 24 hours after the operation.

They commence with shivering, pain in the loins—the pulse rises—the tongue grows dry—the patient still retains his intellects. Now there is some pain in the pubes and left groin—the pulse has a weak beat and intermits, at first, perhaps, once in 40 strokes, then in five or six—there is hiccup, and, if there was no shivering at first, there is shivering now. The belly becomes tympanitic, and, in consequence of the tympanitis, tender. The symptoms go on—generally there is delirium, but occasionally the patient retains his senses to the last, and now death closes the scene.

*On dissection*, you find the intestines distended with air, but no general peritonitis, except just at the lower part of the belly, where the peritonæum is reflected from the rectum to the bladder, and where, perhaps, it is a little inflamed. The cellular membrane of the pelvis is sloughy, inflamed, putrid, and here is the mischief. You may think that peritonitis is common after lithotomy, and the profession generally do think so—but it is not the case. It is this *sloughing of the cellular membrane* that is frequent, and that is constantly mistaken for peritonitis. Don't make this mistake; if you do, if you bleed or purge, the clock runs down the faster. Give, at first, salines, with a little excess of ammonia; then opium, wine, brandy, &c. Adults very seldom recover — children occasionally do so. After presenting these symptoms for two or three weeks, one child recovered, a putrid abscess bursting into the wound. Another child recovered, a slough giving way into the rectum.

*Surgical Treatment of this Affection.* I will mention to you a case.

*Case.* In a patient whom I lithotomized, I used Mr. Blizard's knife, an abominable instrument. I cut through the prostate gland, and all seemed right to the lookers-on. I felt uneasy. In 36 hours there were suspicious symptoms; in 48 hours they were well-marked: there were intermitting pulse, tympanitic belly, manifestly mischief in the cellular membrane, round the neck of the bladder. I introduced my finger into the rectum, and passed Savigny's probe-pointed bistoury up the wound to its left extremity—then I pushed forward the moveable sharp point, divided the coats of the rectum on my finger, and cut outwards with the point resting on my finger, exactly as you operate for fistula ani. The intermissions of the pulse grew less frequent, then ceased, and ultimately the patient recovered.

*Hæmorrhage.* This is another cause of death after lithotomy. I have myself seen three patients die of hæmorrhage. One was a patient of Sir E. Home's; he died when I was house-surgeon. A patient of an eminent lithotomist died. The third was a patient of my own, and in him the hæmorrhage was venous. I have heard of other cases, though I have seen no more.

*Division of Pudic Artery.* Many years ago Sir E. Home operated on a patient for stone. In half an hour after the operation I found the patient apparently dying from profuse hæmorrhage, which was still going on. I brought the patient with his perinæum to the light, and finding that pressure on the pudic artery stopped the bleeding, I tied it, by passing a curved silver needle between it and the bone. The bleeding was arrested and did not return. In a private patient the hæmorrhage was only stopped by an assistant's keeping his finger on the pudic artery for five or six hours together.

Sometimes there is hæmorrhage from the neck of the bladder. The best way under such circumstances, is to introduce the longest finger into the rectum, and press on the neck of the bladder. You should previously pass a canula into the wound; it serves for a point d'appui, and lets out the blood, &c.

### Death from Exhaustion.

*Case.* A patient underwent a tedious operation, the stone breaking, the perinæum deep, the man very fat. He was put to bed, and died with symptoms of exhaustion.

A long operation is always hazardous.

Sometimes the patients fall afterwards into a deep doze or state of coma, and though they sometimes recover, they often die.

Persons affected with organic disease are, of course, not such good subjects for lithotomy, as those who are not so affected. But organic disease of other organs is not near so important as the sound or unsound state of the urinary organs.

*With enlarged Prostate.* I don't think that an enlarged prostate is of any material consequence, for patients who have it do very well. But when the prostate is ulcerated, *never* operate. I have seen it done in three instances, and in each fatally.

*With Chronic Inflammation of the Bladder.* Chronic inflammation of the mucous membrane of the bladder is a serious, but not a fatal objection. If the inflammation of the bladder is acute they die.

*Case* 1. Such a patient, on whom I operated, did pretty well for a week, then fell into a low state, and died. The inflammation extended up the ureter, and muco-purulent matter was found in the pelvis and infundibula. I operated against my own judgment.

*Case* 2. Another patient died four or five weeks after the operation. There was inflammation and suppuration (?) in the cellular membrane *all* round the bladder.

*With Abscess in the Kidney.* If there be abscess in the kidney, the patient is sure to die. Inflammation of the kidney, independent of abscess, confers danger. When there is pain in the loins and across the lower part of the belly—languor and utter listlessness—hands and feet chilly — urine albuminous; when there are these symptoms the case is a bad one for operation. But if the urine contains pus, or any thing approaching to it, the case is still worse. More frequently patients operated on with abscess of the kidney, die after a certain length of time, having apparently recovered from the operation.

*Case.* Sir E. Home operated on such a patient. He said during the operation that something had burst in the loins. He died in three days. On dissection an abscess *was* found to have burst into the cellular membrane, behind the peritonæum and below the kidney.

Generally they die several weeks afterwards with the usual symptoms of suppuration in the kidney.

*Age.* Children generally do well. Persons between 70 and 80 do better than those between 50 and 70. Such is my experience, and such is that of the Norwich Hospital.

*Stones lodging in the Urethra.* Stones sometimes lodge in the urethra. You may take them out. Sometimes they lodge and grow there. I took out two such calculi, the urethra having burst behind the posterior, and having given rise to effusion of urine.

### High Operation.

This is very ancient. Since Cheselden's time the improvement has been introduced of leaving a gum catheter in the urethra and bladder. I have seen it done several times, and it was always what I call a bungling operation. It is more difficult, more hazardous, unjustifiable, except where the stone is too large to be removed by an incision in the perinæum. In such a case I think the recto-vesical operation is more applicable. I have always thought so since the occurrence of the case in which I divided the rectum for sloughing of the cellular membrane in the neighbourhood of the bladder. I lately performed such an operation, and though the stone was smaller than had been anticipated, I extracted it, and might have removed a larger with the greatest ease.

*Case.* In this instance I cut into the bladder in the usual way, then introducing the fore-finger of my right hand into the rectum, I passed one of Savigny's probe-pointed bistouries into the wound, pushed the sharp point forwards, and, dividing the rectum on my finger, drew the bistoury and my finger out together, exactly as in the operation for fistula ani. The opening into the rectum was not made to correspond exactly with the wound in the bladder, but a sort of valve, formed by the parietes of the latter, was interposed between them. I did this to avoid, as far as possible, a vesico-rectal fistula. The patient died after ten days or a fortnight—with a large abscess in the pelvis. I believe, from other cases to which I have already referred, that this abscess existed previous to the operation. The fact is, that I cannot draw any satisfactory inference from this case one way or the other.

*Prostatic Calculi.* Stones sometimes form in the ducts of the prostate gland. They are composed of phosphate of lime, and formed by the secretion of the prostate. Their surface is smooth, like satin. By-and-bye they increase, and are collected in a sort of bag in the prostate; they pass forwards, and some are rejected by the urethra, whilst some get back into the bladder, excite chronic inflammation, and become encrusted with the phosphates.

*Treatment.* Introduce a bougie into the urethra to keep it open and expanded for the passage of a stone. If there are many in a cyst you may extract them by Weiss' forceps. If they are in the bladder, lying and growing there, you may pick them out of it by the same. No medical treatment, that I know of, will prevent the formation of prostatic calculi.

### Lithotomy in the Female.

I have already explained how women are less liable to the formation of calculi in the bladder than men. The causes are briefly these:—their large urethra—temperate mode of life—and absence of enlarged prostate to retain the calculus in the bladder.

*Symptoms.* As in the male, there is pain referred to the extremity of the urethra, and the symptoms in all other respects resemble those in the male.

*Operation.* You may remove the stone by a cutting instrument. Introduce a straight staff through the urethra into the bladder, then along it a knife, or, what is better, a bistoire cachée;—turn the cutting edge of the bistoire downwards and to one side, and withdraw it, by which you divide the neck of the bladder and urethra. This operation is not dangerous, but women are subject afterwards to incontinence of urine. Mr. Hey recommended the introduction of a sponge into the vagina, after the operation, to keep the sides of the wound together. I tried this in a female child in the hospital, yet nevertheless there was incontinence of urine.

*Division upwards.* It has been proposed to divide merely the membranous part of the urethra upwards towards the pubes, and then to dilate the passage without cutting into the cellular membrane. I did this in one instance, and, although the patient could not retain her urine so well as before, there was no incontinence of it afterwards. Mr. Hodgson has seen several cases in which this plan succeeded, and I believe that, when you employ a cutting instrument, this is the best method.

*Dilatation.* This was commonly employed a century ago, but it fell into disuse and was forgotten. It was revived in this manner. Mr. Thomas was called to a lady, who, by some means or other, got a silver tooth-pick into her bladder through the urethra. Mr. Thomas introduced a sponge-tent into the urethra, then got in his finger, and by means of that and the forceps he extracted the tooth-pick. Sir Astley Cooper next removed a stone in the same manner.

A lady applied to me with stone in the bladder. I introduced three sponge-tents in succession, and could easily insert my finger. I extracted the stone.

Mr. Weiss has invented a dilator infinitely better than the sponge-tent.

By it you may dilate the urethra, so as to extract a pretty large calculus. For one of moderate size you may dilate gradually for 24 hours. A surgeon did it suddenly, and the patient nearly died of what he thought was peritonitis. In my cases the patients did not ultimately suffer from incontinence of urine, though they had some for a time after the operation. The same thing occurred, I believe, in the cases of Sir Astley Cooper and others. I have heard, however, of cases, in which there was incontinence left. On the whole I prefer dilatation to cutting.

### Lithontrity.

Mr. Key demonstrated the facility of using a straight staff in the operation of lithotomy; and much about the same time the French surgeons employed straight instruments in the treatment of stricture of the urethra. The French surgeons have now arrived at the use of a straight instrument which shall seize the stone and grind it in the bladder. I never myself employed this instrument, and therefore can only make a few general observations on the operation of lithontrity.

In this operation there is no cutting, which to a patient is a great point—it is not much of an operation—the patient generally suffers little. This is the *favourable* side of the question.

But sometimes there is considerable pain—the operation is not always done at once—if extreme caution is not used a fragment may be left behind in the bladder—it is not applicable to large stones, or large prostates. This is the *unfavourable* side of the question.

Although the operation is generally safe, it is not always so. All the circumstances which render the common operation hazardous render this hazardous also. Yet, after all, it is a great improvement in modern surgery. In ordinary cases it is attended with little danger, and not much pain. The patient does not always drive the operation off, like lithotomy, and that is a great recommendation. Unfortunately this operation does not relieve us of those cases in which we dread the lateral operation; it is only applicable to a limited number of cases. In those to which it is applicable it is a very nice operation; but a man must devote himself almost entirely and exclusively to its performance.

---

